# Headache-attributed burden among children and adolescents in Nepal: a national schools-based cross-sectional study

**DOI:** 10.1186/s10194-026-02472-2

**Published:** 2026-08-01

**Authors:** Rajeev Ojha, Ragesh Karn, Bikram Prasad Gajurel, Reema Rajbhandari, Niraj Gautam, Bikash Deo, Aakarshan Timilsina, Anushka Adhikari, Ravi Raj Timasina, Gaurav Nepal, Derya Uludüz, Tayyar Şaşmaz, Bengü Nehir Buğdaycı Yalçın, Andreas Kattem Husøy, Timothy J. Steiner

**Affiliations:** 1 Department of Neurology, Tribhuvan University Teaching Hospital, Kathmandu, Nepal; 2 Department of Medicine, Tribhuvan University Teaching Hospital, Kathmandu, Nepal; 3 Department of Medicine, Lamjung District Hospital, Besisahar, Nepal; 4 Department of Psychiatry, Gandaki Medical College, Pokhara, Nepal; 5 Department of Neurology, Faculty of Medicine, Istanbul Atlas University, Istanbul, Turkey; 6 Department of Public Health, Mersin University School of Medicine, Mersin, Turkey; 7 Department of Neuromedicine and Movement Science, Norwegian Centre for Headache Research (NorHead), Norwegian University of Science and Technology, Trondheim, Norway; 8 Department of Neurology and Clinical Neurophysiology, St Olavs University Hospital, Trondheim, Norway; 9 Department of Neurology, University of Copenhagen, Copenhagen, Denmark; 10 Division of Brain Sciences, Imperial College London, London, UK; 11 Department of Neuromedicine and Movement Science, Norwegian University of Science and Technology (NTNU), Edvard Griegs gate 8, Trondheim, Norway

**Keywords:** Headache disorders, Migraine, Tension-type headache, Medication-overuse headache, Undifferentiated headache, Epidemiology, Children, Adolescents, Burden of disease, Schools-based survey, Nepal, South East Asia region, Global Campaign against Headache

## Abstract

**Background:**

We recently published estimates of 1-year headache prevalence among children (6–11 years) and adolescents (12–17) in Nepal. Adjusted for age and gender, these were 83.9% for all headache, 39.5% for migraine, 31.7% for undifferentiated headache (UdH), 10.0% for tension-type headache (TTH), 0.3% for probable medication-overuse headache (pMOH) and 1.9% for other headache on ≥ 15 days/month (other H15+). Here we present estimates of attributed burden.

**Methods:**

We followed the Global Campaign’s standardised protocol. In nine schools representative of the country, the child and adolescent versions of the structured HARDSHIP questionnaire were completed by pupils in class under supervision. Headache diagnoses followed ICHD-3, except for UdH, defined here as mild or moderate headache with usual duration < 1 h. Burden enquiries were in multiple domains, with timeframes of 4 weeks and 1 day, the latter based on reports of headache yesterday (HY).

**Results:**

There were 2,352 participants (1,040 children [44.2%]; 1,312 adolescents [55.8%]) from 2,360 eligible (participating proportion 99.7%). Symptom burden was expressed as moderate headache on an average of 2.9 days/4 weeks, lasting for 2.0 h. Mean proportion of time in ictal state (pTIS) was 1.0%, but much higher among those with pMOH (11.0%) or other H15+ (9.4%). Except for pMOH, only one third of headache episodes were medicated. Lost school time was 3% estimated from recall over the preceding 4 weeks, with similar time lost from other (social and leisure) activities. Actual absences from school yesterday because of HY were more (4.5%), suggesting losses were underestimated by recall, but, since recorded absences yesterday failed to capture absences on days preceding weekends or holidays, true losses might have been 5–6%. More than one fifth (21.7%) of parents lost time from their own work. Both emotional impact and quality-of-life (QoL) scores were sensitive to headache (and headache type), showing gradients of negative impact (pMOH and other H15+ > migraine > TTH and UdH).

**Conclusions:**

Symptom burden appeared to be relatively modest except for pMOH and H15+, but measures of impaired participation (including lost time from school), emotional impact and QoL all indicated material impact. Parents, teachers, health-care providers and policy makers should be aware of this.

## Introduction

We recently published estimates of 1-year headache prevalence among children (6–11 years) and adolescents (12–17 years) in Nepal [1]. Adjusted for age and gender, estimates were 83.9% for all headache, 39.5% for migraine, 31.7% for undifferentiated headache (UdH), 10.0% for tension-type headache (TTH), 0.3% for probable medication-overuse headache (pMOH) and 1.9% for other headache on ≥15 days/month (other H15+). These are the headache types of public-health importance, and, in these age groups, of importance also through their potential impact on education and social development. Here we present estimates of attributed burden, derived from data collected at the same time from the same participants.

The study was attended by difficulties in diagnosing the many cases of short-duration headache (<1 h). Similar difficulties, leading to unfeasibly high estimates for migraine (including probable migraine) had been encountered in studies in Benin [2], Zambia [3] and Georgia [4]. In an earlier study in Turkey, we introduced the concept of UdH, defined as mild headache lasting <1 h and believed to be expressions by the still-developing brain of what might later become migraine or TTH [5]. This concept was subsequently applied in studies in Ethiopia [6], Lithuania [7], Mongolia [8] and Iran [9]. In Benin [2], Zambia [3] and Georgia [4], we proposed a modification of this definition to include moderate headache, but retaining the essential criterion of short duration. Diagnostic distinction, we argued, should not depend on the highly subjective differentiation, especially in these young age groups, between mild and moderate headache. We adopted this modified definition here in Nepal [1].

The purposes of this study, the first in South East Asia Region (SEAR) of the series of similar schools-based studies conducted within the Global Campaign against Headache [10], were to enhance understanding of the headache burden among children and adolescents in Nepal and worldwide, and to inform public-health and educational policies in Nepal.

## Methods

This was a cross-sectional survey conducted in schools across Nepal following the programme’s standardised protocol [11]. Pupils completed a structured questionnaire in class, under the supervision of school staff.

The methodology has been described in detail previously [1], and therefore is only summarised here.

### Ethics and approvals

The research was conducted in accordance with the Declaration of Helsinki.

Approvals were obtained from the Institutional Review Committee of Tribhuvan University Institute of Medicine (169 [6-11]F2 078/079) along with necessary authorisations from academic and administrative authorities and permissions from principals and class teachers of each school.

All participants gave oral consent before enrolment, with continuing consents evidenced by active completion of the questionnaires. In view of the non-interventional nature of the study, neither the Institutional Review Committee nor the academic and administrative authorities requested prior parental consent.

Data were recorded anonymously, and managed in accordance with local data protection regulations.

### Sampling and recruitment

Data collection was from March to November 2022.

Adopting a cluster-sampling methodology, we included nine schools representing a mix of urban and rural populations across the socioeconomic range and from altitudes 70 to 2,860 m above sea level. The schools were in nine districts, in the administrative centres of six of Nepal’s seven provinces: Biratnagar and Lukla in province 1, Janakpur in province 2, Kathmandu and Dhulikhel in province 3, Pokhara and Besisahar in province 4, Butwal in province 5 and Dunai in province 6.

All pupils aged 6–17 years present on survey days were invited to participate.

### Data collection

We used the child and adolescent versions of the Headache-Attributed Restriction, Disability, Social Handicap and Impaired Participation (HARDSHIP) questionnaire [11] translated into Nepali following the Global Campaign’s translation guidelines for lay documents [12]. These were distributed to the pupils in class, who completed them under supervision by the researchers and class teachers. Guidance and clarification were provided when needed.

Demographic enquiry was followed by headache screening and diagnostic questions (the latter based on ICHD-3 criteria [13] except for UdH), then by enquiries into attributable burden (symptom burden, medication use, impaired participation [lost time from school and other activities], lost parental worktime, emotional impact and quality of life [QoL]). The timeframes of these enquiries were the preceding 4 weeks (28 days) and 1 day. The latter circumvented recall error through questions related to headache on the day prior to the survey day (headache yesterday [HY]), whenever this was reported to have occurred.

### Headache diagnoses

Diagnoses were made hierarchically, applying the HARDSHIP algorithm [14]. Pupils reporting headache on ≥14 days in the previous 28 days (assumed to equate to ≥15 days in the previous 30 days) were diagnosed as pMOH when also reporting medication use on ≥14 days, and otherwise classified as “other H15+”. Among the remainder, we first identified UdH, then definite migraine, definite TTH, probable migraine and probable TTH. Cases remaining undiagnosed were labelled “unclassified headache”.

In the analyses, definite and probable migraine were combined, as were definite and probable TTH.

### Analysis

Analyses were performed at University of Mersin, Turkey, and at the Norwegian Centre for Headache Research (NorHead).

Headache frequency and acute medication intake were reported as days/4 weeks. Headache intensity was reported as “mild”, “moderate” or “severe”, which we converted to a numerical scale of 1–3, treating this as continuous. Duration of headache was reported categorically, as <1, 1–2, 2–4 or >4 h, which we analysed by taking the mid-points (0.5, 1.5, 3 and 8 respectively, assuming a range of 4–12 h for the last). We calculated proportion of time in ictal state (pTIS) as the product of frequency and duration. We estimated lost health at individual level (%) as the product of pTIS and the disability weight (DW) allocated in the Global Burden of Disease (GBD) study to the ictal state of the headache type (migraine, TTH or pMOH) on a scale 0–1, where 0 indicated no health loss and 1 a health loss equivalent to being dead [15].

To estimate lost school time in the preceding 4 weeks, we counted complete absences (not going to school) as a whole day lost and partial absences (leaving school early) as a half-day lost, and calculated proportions from the sum of these over a denominator of 20. We counted days of limited activity (defined as “could not do things you wanted to because of your headaches”), and calculated proportions presuming a denominator of 28. We counted parents who had, according to the pupils, lost time from their own work during the same period (regardless of quantity). In analyses based on HY, we calculated actual proportions who had missed school yesterday because of HY, comparing these with predicted proportions (the product of number affected by headache and mean reported days [divided by 20] lost per pupil over the preceding 4 weeks).

Responses to questions addressing concentration, emotional impact and QoL were all on a 4-point Likert scale (“never”, “sometimes”, “often”, “always”) [11], also referring to the preceding 4 weeks. We scored these 0–3, and summed them to generate an emotional impact score (including concentration; potential range 0–18, high being adverse) and a QoL score (0–36, low being adverse).

In descriptive statistics we used means, standard deviations (SDs) and standard errors (SEs) for continuous variables and proportions (%) with 95% confidence intervals (CIs) for categorical data. We used Kruskal-Wallis with post hoc Dunn’s test to evaluate group-differences for continuous variables and binary logistic regression for binary outcomes.

Population-based estimates were derived by factoring in prevalence.

In all analyses, we set significance at *p* < 0.05.

## Results

From 2,360 eligible pupils, there were 2,352 participants (1,040 children [44.2%]; 1,312 adolescents [55.8%]; 1,269 males 54.0%; 1,083 females [46.0%]; participating proportion 99.7%). Their distributions between urban (55.4%), semirural (20.2%) and rural areas (24.4%) of Nepal, and across altitudes (44.6% below 1,000 m, 31.0% between 1,000 and 2,000 m, and 24.5% above 2,000 m), have been reported previously [1]. Also reported previously (above, and [1]), but reiterated here because they are used in population-level burden estimates, age- and gender-adjusted estimates of prevalence were 83.9% for all headache, 39.5% for migraine, 10.0% for TTH, 31.7% for UdH, 0.3% for pMOH and 1.9% for other H15+, with 0.6% unclassified. HY was reported by 488 participants (20.7%).

**Table 1:** Symptom burden by headache type and overall

Headache type	Pupils affected (*n*)	Headache frequency days/4 weeks (mean±SD)	Headache duration hours (mean±SD)	Estimated pTIS % (mean±SE)	Headache intensity scale 1–3^1^ (mean±SD)
**Episodic headaches**					
Migraine	940	3.0±2.5	3.0±2.6	1.4±0.1	2.5±0.5
TTH	246	2.3±2.4	2.2±1.9	0.7±0.1	2.1±0.6
UdH	755	1.9±2.1	0.5± < 0.1	0.1± < 0.1	1.9±0.4
**Headache on ≥ 15 days/month**					
pMOH	7	21.7±5.9	3.6±3.1	11.0±3.1	2.3±0.5
Other H15+	47	17.9±4.6	3.5±3.2	9.4±1.3	2.4±0.6
**All headache** ^**2**^	2,008	2.9±3.6	2.0±2.3	1.0±0.1	2.2±0.6

SD: standard deviation; SE: standard error; pTIS: proportion (%) of time in ictal state; TTH: tension-type headache; UdH: undifferentiated headache; pMOH: probable medication-overuse headache; H15+: headache on ≥15 days/month; ^1^equating 1–3 to “mild”, “moderate” and “severe”, and treating as continuous data; ^2^includes unclassified headache

**Table 2:** Symptomatic medication use by headache type and overall

Headache type	Pupils responding *n*	Medication use days in preceding 4 weeks (mean ± SD)	Headache frequency days/4 weeks (from Table 1)
**Episodic headaches**			
Migraine	940	1.2±1.7	3.0
Tension-type headache	246	0.7±1.4	2.3
Undifferentiated headache	755	0.6±1.1	1.9
**Headache on ≥ 15 days/month**			
Probable medication-overuse headache	7	20.4±5.7	21.7
Other headache on ≥ 15 days/month	47	2.7±4.0	17.9
**All headache** ^**1**^	2,008	1.0±2.0	2.9

^1^Includes unclassified headache

### Symptom burden

Measures of symptom burden are shown in Table 1. Headache frequency was, inevitably, skewed by H15+, but the overall mean was 2.9 days/4 weeks. Mean duration was 2.0 h, with SD = 2.3 indicating that this was also skewed. Mean pTIS was 1.0%. Mean intensity was 2.2 (moderate). On all of these measures, migraine was associated with higher symptom burden than TTH, and TTH with higher burden than UdH. pMOH and other H15+ were not only much more frequent (21.7 and 17.9 days/4 weeks respectively), but also longer lasting than the episodic headaches, with very much higher estimates of pTIS (11.0% and 9.4% respectively). Intensity of these headache types was similar to that of migraine.

Table 2 shows that, for migraine, TTH and UdH, only about one third of headache days were medicated. As expected, symptomatic medication was taken on almost all days of pMOH. For other H15+, frequency of medication (2.7 days/4 weeks) was very much at odds with frequency of headache (17.9 days/4 weeks), notwithstanding the relatively high duration (mean 3.5 h) and intensity (2.4).

**Table 3:** Headache-attributed lost health at individual and population levels by headache type

Headache type	Disability weight (from [15])	Age- and gender-adjusted prevalence (from [1]) %	Mean pTIS (from Table 1) %	Lost health %	
				Individual^1^ (mean)	Population^2^
Migraine	0.441	39.5	1.4	0.6	0.2
Tension-type headache	0.037	10.0	0.7	0.03	0.003
Probable medication-overuse headache	0.223	0.3	11.0	2.5	0.007

pTIS: proportion of time in ictal state; ^1^calculated as the product of mean pTIS and disability weight; ^2^calculated as the product of mean individual lost health and prevalence (with apparent discrepancies due to rounding)

Table 3 shows the health loss attributed to migraine, TTH and pMOH at individual and population levels. This measure, corresponding to reduction in healthy life expectancy, is estimated as pTIS*DW for the ictal state, but DWs were available from GBD only for these three headache types [15]. At individual level, pMOH was associated with by far the greatest health loss (2.5%), four times that attributable to migraine (0.6%). At population level, however, pMOH had little impact because of its very low prevalence. At this level, health loss attributed to migraine was 0.2%, with TTH having almost no impact.

Impaired participation in school and other activities (social, leisure and play) is shown in Table 4. Among those reporting any headache, 0.6 days/4 weeks of missed school were attributed to it, an estimated loss of 3% of 20 days. Children lost rather more (0.8 days/4 weeks, or 4%), but SDs were large. Reported pMOH-attributed losses were far greater (3.4 days/4 weeks, or 17%), but from *n* = 7. Other H15+ was responsible for 1.8 missed days/4 weeks (9%), 3.0 (15%) among children and 1.3 (6.5%) among adolescents. For the episodic headaches, days of limited activities were similar in number to missed school days, or rather more, but from a presumed denominator of 28 days rather than 20 (see Methods [Analysis]). pMOH reportedly had far greater impact, but again from *n* = 7, while other H15+ was blamed for 4.4 days of limited activities (16% of all days), more than twice as many among adolescents (5.1, or 18%) as among children (2.4, or 9%).

**Table 4:** Lost time from school and other activities because of headache in the preceding 4 weeks, at individual level, by headache type and overall, and by age group

Headache type	Headache frequency (from Table 1) days/4 weeks (mean±SD)	Days of lost school	Days of limited activity
		days/4 weeks/affected pupil (mean ± SD)	
**Episodic headaches**			
Migraine children adolescents	3.0±2.5 3.2±2.6 2.8±2.5	0.8±1.4 1.0±1.4 0.7±1.4	1.2±1.5 1.0±1.4 1.3±1.6
Tension-type headache children adolescents	2.3±2.4 1.9±2.2 2.4±2.4	0.5±1.2 0.5±1.5 0.5±1.0	0.7±1.2 0.4±0.9 0.9±1.3
Undifferentiated headache children adolescents	1.9±2.1 1.9±2.1 1.9±2.1	0.4±0.9 0.4±0.9 0.4±0.9	0.6±1.1 0.5±1.0 0.7±1.2
**Headache on ≥ 15 days/month**			
Probable medication-overuse headache children adolescents	21.7±5.9 22.0±5.1 21.0±9.9	3.4±4.4 2.6±3.5 5.3±7.4	12.6±11.5 9.2±11.1 21.0±9.9
Other headache on ≥ 15 days/month children adolescents	17.9±4.6 18.8±6.0 17.6±3.9	1.8±3.4 3.0±5.2 1.3±2.4	4.4±5.7 2.4±3.4 5.1±6.2
**All headache** ^**1**^ children adolescents	2.9±3.6 2.9±3.6 2.8±3.6	0.6±1.4 0.8±1.5 0.6±1.3	1.0±1.9 0.9±1.7 1.2±2.1

^1^Includes unclassified headache

Table 5 shows actual lost days from school yesterday because of HY along with absences predicted from prevalence and mean reported lost days/4 weeks. There were only 18 actual absences from migraine, four from UdH and none from TTH, pMOH or other H15+ (for all of which – especially pMOH – relatively few pupils reported HY). Nevertheless, overall, actual absences were more than predicted (Table 5), which might indicate a tendency to underestimate in recall over 4 weeks (this is discussed later).

**Table 5:** Lost school days yesterday because of headache yesterday (HY), at individual level, by headache type and overall, with predictions based on recalled lost days/4-week

Diagnosed headache type	Pupils affected *n*	Proportion reporting HY *n* (% of those with headache type)	Missed school day yesterday *n* (% of those reporting HY)	
			Actual	Predicted
**Episodic headaches**				
Migraine	940	272 (28.9)	18 (6.6)	11 (4.0)
Tension-type headache	246	51 (20.7)	0 (0.0)	1 (1.9)
Undifferentiated headache	755	121 (16.0)	4 (3.3)	2 (1.7)
**Headache on ≥ 15 days/month**				
Probable medication-overuse headache	7	5 (71.4)	0 (0.0)	1 (20.0)
Other headache on ≥ 15 days/month	47	37 (78.7)	0 (0.0)	3 (8.1)
**All headache** ^**1**^	2,008	488 (24.3)	22 (4.5)	15 (3.1)

^1^Includes unclassified headache

Table 6 shows that parental lost time from work was a common occurrence, although amount of lost time was not recorded since younger participants might not be able to judge this. Overall, 21.7% of parents lost time, those of children (28.7%) more than those of adolescents (17.0%). A similar or larger differential was seen for all headache types except other H15+ (*n* = 18).

Table 7 statistically compares the measures of impaired participation. What it shows is that, despite the diagnostic uncertainties surrounding migraine [1], pupils classified as having migraine reported clearly greater impacts on all of these measures than those classified as having TTH or UdH.

Table 8 shows the same with regard to emotional impact and QoL scores: pupils classified as having migraine reported clearly higher impacts than those classified as having TTH or UdH. Across all headache types, emotional impact scores were on a gradient: pMOH and other H15+ > migraine > TTH = UdH. Despite a low baseline score (24/36 among those with no headache), QoL scores showed much the same, with impact reflected in lower scores: pMOH < other H15+ < migraine < TTH = UdH.

**Table 6:** Parents’ lost work in the preceding 4 weeks, by pupils’ headache type and overall, and by age group

Headache type	Pupils affected *n*	Headache frequency days/4 weeks (from Table 1)	Parent missed work *n* (% of pupils with headache type)
**Episodic headaches**			
Migraine children adolescents	940 395 545	3.0 3.2 2.8	274 (29.1) 148 (37.5) 126 (23.1)
Tension-type headache children adolescents	246 76 170	2.3 1.9 2.4	39 (15.9) 15 (19.7) 24 (14.1)
Undifferentiated headache children adolescents	755 308 447	1.9 1.9 1.9	94 (12.5) 55 (17.9) 39 (8.7)
**Headache on ≥ 15 days/month**			
Probable medication-overuse headache children adolescents	7 5 2	21.7 22.0 21.0	4 (57.1) 3 (60.0) 1 (50.0)
Other headache on ≥ 15 days/month children adolescents	47 13 34	17.9 18.8 17.6	18 (38.3) 5 (38.5) 13 (38.2)
**All headache** ^**1**^ children adolescents	2,008 804 1,204	2.9 2.9 2.8	436 (21.7) 231 (28.7) 205 (17.0)

^1^Includes unclassified headache

**Table 7:** Comparisons of headache types for measures of impaired participation (Kruskal-Wallis with post-hoc Dunn’s test)

Lost school days/4 weeks						
	*n* ^1^	mean±SD	TTH	UdH	pMOH	Other H15+
Migraine	937	0.8±1.4	**< 0.001**	**< 0.001**	0.56	0.47
TTH	246	0.5±1.2		1.00	0.07	**0.001**
UdH	751	0.4±0.9			0.05	**< 0.001**
pMOH	7	3.4±4.4				1.00
Other H15+	47	1.8±3.4				
**Lost days/4 weeks from other activities**						
Migraine	935	1.2±1.5	**< 0.001**	**< 0.001**	**0.006**	**< 0.001**
TTH	246	0.7±1.2		1.00	**< 0.001**	**< 0.001**
UdH	754	0.6±1.1			**< 0.001**	**< 0.001**
pMOH	7	12.6±11.5				0.69
Other H15+	47	4.4±5.6				
**Lost parental worktime** n^1^ (%) of parents of pupils with headache type reported to have lost time in preceding 4 weeks						
Migraine	274	29.1%	**< 0.001**	**< 0.001**	0.54	0.67
TTH	39	15.9%		0.65	0.09	**0.005**
UdH	94	12.5%			**0.03**	**< 0.001**
pMOH	4	57.1%				0.88
Other H15+	18	38.3%				

TTH: tension-type headache; UdH: undifferentiated headache; pMOH: probable medication-overuse headache; H15+: headache on ≥ 15 days/month; ^1^values reflect that not all pupils responded to every enquiry; significant p-values are emboldened

**Table 8:** Emotional impact and quality of life scores by headache type

Headache type	Pupils responding *n* ^1^	Score mean ± SD	Between-type significance (Kruskal-Wallis with post-hoc Dunn’s test)			
			TTH	UdH	pMOH	Other H15+
**Emotional impact** (0–18; higher scores adverse)						
Migraine	937	7.4±2.6	**< 0.001**	**< 0.001**	0.93	0.05
TTH	245	6.2±2.7		1.00	0.07	**< 0.001**
UdH	754	6.0±2.3			**0.03**	**< 0.001**
pMOH	7	8.9±2.8				1.00
Other H15+	47	8.8±3.3				
All headache^2^	2,003	6.8±2.7				
**Quality of life** (0–36; lower scores adverse)						
No headache	340	24.0±5.0	**< 0.001** compared to all headache types			
Migraine	937	19.9±5.2	**< 0.001**	**< 0.001**	0.17	0.05
TTH	245	21.7±5.1		1.00	**0.01**	**< 0.001**
UdH	754	21.7±4.6			**0.01**	**< 0.001**
pMOH	7	15.7±4.6				1.00
Other H15+	47	17.9±6.0				
All headache^2^	2,003	20.8±5.1				

TTH: tension-type headache; UdH: undifferentiated headache; pMOH: probable medication-overuse headache; headache on ≥ 15 days/month; ^1^values reflect that not all pupils responded to every enquiry; ^2^includes unclassified headache; significant p-values are emboldened

## Discussion

This study in Nepal was important in the Global Campaign’s schools-based series estimating the global burden of headache among children and adolescents, since it was the first in SEAR.

Symptom burden was expressed as moderate headache occurring on an average of 2.9 days/4 weeks and lasting for 2.0 h. Mean pTIS was 1.0%, not high except among those with pMOH (11.0%) or other H15+ (9.4%). Except for pMOH, only one third of headache episodes were medicated, which was unsurprising in view of the short duration of most headaches. What was surprising was that those with other H15+, reporting headache on 17.9 days/4 weeks of moderate-to-severe intensity and mean duration of 3.5 h, used symptomatic medication only on 2.7 days/4 weeks (15.0% of attacks). Other H15+ was not further diagnosed, but would have included any cases (if there were any) of chronic migraine and chronic TTH.

Lost school time – perhaps the most concerning consequence of headache among young people – was an estimated 3%, reflecting the modest symptom burden, but 9% among pupils (*n* = 47) with other H15+ (again making the low usage of medication rather surprising). Similar amounts of time (but 16% among those with other H15+) were reportedly lost from other activities (social, leisure and play – important for social development). These estimates were based on recall over the preceding 4 weeks. Actual absences yesterday because of HY – an observation not dependent on recall – were more than predicted from prevalence and mean reported lost days/4 weeks (4.5% *versus* 3.1%), suggesting that recall tended towards underestimation. This is particularly true since recorded actual absences were themselves an underestimate (by at least 20%), failing to capture absences on days immediately preceding weekends or holidays (which were never survey days). Impact on schooling might, therefore, have been significantly higher than these findings suggest: true losses of perhaps 5–6%. More than one fifth (21.7%) of parents lost time from their own work, an often-unrecognised consequence of child and adolescent headache. Decreasing dependence, offsetting increasing prevalence, probably explained the diminution among adolescents.

Both emotional impact and QoL scores were sensitive to headache (and headache type), showing gradients of negative impact (pMOH and other H15+ > migraine > TTH and UdH).

Our earlier estimates of prevalence encountered diagnostic uncertainties among the many participants reporting headache of usual duration <1 h [1]. Similar uncertainties had occurred in studies using the same questionnaire in Benin [2], Zambia [3], Georgia [4] and, to a lesser extent, Ethiopia [6]. We applied the modified definition of UdH developed in those studies (that is, including mild *and* moderate headache), which accordingly was diagnosed in 31.7% of participants. But still we found a very high proportion meeting criteria for migraine (39.5% after adjustment for age and gender). Despite these uncertainties, most estimates of the *headache*-attributed burdens remain valid, at least insofar as participants’ responses were reliable. The exception was lost health, for which estimates incorporated diagnosis-specific DWs [15].

The measures of impaired participation (lost school time, lost time from other activities and lost parental work time), and the emotional impact and QoL scores, all indicated that, despite their high proportion, participants classified as having migraine were describing an illness with clearly higher detrimental impacts than TTH or UdH. This lends credibility to the diagnostic classification.

Those classified as having other H15+ appeared to fare much worse (in terms of measurable burden) than those with pMOH, despite rather lower reported headache frequency. A speculative – and thought-provoking – explanation would be that this was a consequence of *not* using medication to mitigate symptoms. Numbers, however, were low. Reassuringly, because of the low usage of medication, the estimated 1.9% with other H15+ did not appear to be at immediate risk of developing pMOH. It is worth noting that the prevalences of pMOH and other H15+ among adults in Nepal have been estimated, in a Global Campaign study, as 2.1% and 5.3% respectively [16]. (It might also be noted that, in the same study, the estimated prevalence of migraine was 34.1%, partly explained by a strong association with residence at high altitude [16]).

Overall, these findings indicate negative impacts of headache in these age groups that are greater than might be expected from reported symptom burden. If any headache on a particular day is associated with a probability of missing school in the range 5–6% (recorded absences yesterday being 4.5%, but with > 20% unrecorded), this is, or should be, of concern to both health and educational policies. That over one fifth of parents lose at least some time from their work each 4 weeks should probably be of concern to employers, and to those with broader interests in or responsibilities for productivity and economic targets. Emotional impact and QoL scores both lack intuitive meaning, so are difficult to interpret. The latter, however, started from a low baseline of 24/36 (in those with no headache), yet was significantly reduced among those with any type of headache, and further reduced (to <20) among those with migraine, pMOH or other H15+.

There are only three similar studies reporting headache-attributed burden in these age groups while applying the same definition of UdH: in Benin [17], Zambia [18] and Georgia [19]. Comparisons between these three studies have already been made [19], and these do not bear repetition here. However, comparison of Nepal with Georgia – although ethnically and culturally very different – may be of more interest than comparisons with countries in sub-Saharan Africa (Benin and Zambia). Headache frequency was lower in Nepal than in Georgia (3.0 *versus* 4.4 days/4 weeks [19]), with similar duration and rather higher intensity. These differences, leading to lower pTIS in Nepal than in Georgia (1.0% *versus* 1.5% [19]), were partly driven by lower prevalence of UdH in Georgia, but higher prevalences of pMOH and other H15+. In both countries, a majority of headache episodes, of all types except pMOH, were unmedicated. Lost school days were very similar in the two countries (both as recalled over 4 weeks and according to actual absences with HY); lost days from other activities were rather lower in Nepal than in Georgia (1.0 *versus* 1.4 days/4 weeks [19]); both measures had large SDs. More parents in Nepal (21.7%) than in Georgia (9.4% [19]) lost time from their own work, for reasons more likely to be cultural than disease-related. Finally, emotional impact scores showed very similar impacts of each headache type, as did QoL scores, taking into account the lower QoL baseline score in Nepal (24.0) than in Georgia (28.5) [19]. In summary, such differences as there are between these countries are explained more by differences in prevalence of each headache type than in the burdens attributable to each.

The strengths of this study were in the use of standardised methodology, a large and representative sample (*N* = 2,352) and very high participating proportion (99.7%) [1]. There were the inherent limitations of cross-sectional surveys and reliance upon information gathered from children, although uncertainties in recall over the past 4 weeks were countered by separate enquiry into HY. The diagnostic uncertainties did not negate attribution of burden to *headache*.

## Conclusion

These findings complement those of our previous study of headache prevalence among children and adolescents in Nepal [1], while being more informative to health and educational policies. Measures of impaired participation, emotional impact and impairment of QoL all indicated greater impacts than might be expected from reported symptom burden. Attention needs to be paid (by parents, teachers, health-care providers and policy makers) to how much time is lost from school because of headache.

## Data Availability

The original data are held at Tribhuvan University Teaching Hospital, Kathmandu, Nepal, and the analytical set at University of Mersin, Mersin, Turkey, and at Norwegian University of Science and Technology, Trondheim, Norway. When analyses are completed, anonymised data will be available on request for academic purposes, in line with the policy of the Global Campaign against Headache.

## Abbreviations

CI: confidence interval
d/m: days/month
DW: disability weight
GBD: Global Burden of Disease (study)
H15+: headache on ≥15 days/month
HARDSHIP: Headache-Attributed Restriction, Disability, Social Handicap and Impaired Participation (questionnaire)
HY: headache yesterday
ICHD: International Classification of Headache Disorders
LTB: *Lifting The Burden*
MOH: medication-overuse headache
NorHead: Norwegian Centre for Headache Research
NTNU: Norwegian University of Science and Technology
pMOH: probable MOH
pTIS: proportion of time in ictal state
QoL: quality of life
SD: standard deviation
SE: standard error
SEAR: South East Asia Region
TTH: tension-type headache
UdH: undifferentiated headache
